# Upgradation of methane in the biogas by hydrogenation of CO_2_ in a prototype reactor with double pass operation over optimized Ni-Ce/Al-MCM-41 catalyst

**DOI:** 10.1038/s41598-023-36425-5

**Published:** 2023-06-08

**Authors:** Pichawee Aieamsam-Aung, Atthapon Srifa, Wanida Koo-Amornpattana, Suttichai Assabumrungrat, Prasert Reubroycharoen, Phorndranrat Suchamalawong, Choji Fukuhara, Sakhon Ratchahat

**Affiliations:** 1 Energy Research Institute, Chulalongkorn University, Bangkok, 10330 Thailand; 2 Center of Excellence on BCG Towards Sustainable Development, Chulalongkorn University, Bangkok, 10330 Thailand; 3 Department of Chemical Engineering, Faculty of Engineering, Mahidol University, Nakhon Pathom, 73170 Thailand; 4 Center of Excellence in Catalysis and Catalytic Reaction Engineering, Department of Chemical Engineering, Faculty of Engineering, Chulalongkorn University, Bangkok, 10330 Thailand; 5 Department of Chemical Technology, Faculty of Science, Chulalongkorn University, Bangkok, 10330 Thailand; 6 Marine Department, Merchant Marine Training Centre, 1278 Yotha Road, Talard Noi, Samphanthawong, Bangkok, 10100 Thailand; 7 Department of Applied Chemistry and Biochemical Engineering, Graduate School of Engineering, Shizuoka University, Shizuoka, 432-8561 Japan

**Keywords:** Environmental sciences, Energy science and technology, Engineering, Nanoscience and technology

## Abstract

The upgradation of methane in biogas by hydrogenation of CO_2_ has been currently recognized as a promising route for efficient full utilization of renewable biogas with potential benefits for storage of renewable hydrogen energy and abatement of greenhouse gas emission. As a main constituent of biogas, CO_2_ can act as a backbone for the formation of additional CH_4_ by hydrogenation, then producing higher amounts of biomethane. In this work, the upgradation process was investigated in a prototype reactor of double pass operation with vertical alignment using an optimized Ni-Ce/Al-MCM-41 catalyst. The experimental results show that the double pass operation that removes water vapor during the run can significantly increase CO_2_ conversion, resulting in higher CH_4_ production yield. As a result, the purity of biomethane increased by 15% higher than a single pass operation. In addition, search for optimum condition of the process was carried out within an investigated range of conditions including flowrate (77–1108 ml min^−1^), pressure (1 atm–20 bar), and temperature (200–500 °C). The durability test for 458 h was performed using the obtained optimum condition, and it shows that the optimized catalyst can perform excellent stability with negligible influence by the observed change in catalyst properties. The comprehensive characterization on physicochemical properties of fresh and spent catalysts was performed, and the results were discussed.

## Introduction

Currently, renewable energy resources such as solar energy, wind, geothermal, hydropower, solid biomass, liquid biofuels, and biogas have been widely recognized as potential candidates for substituting fossil-based energy resources. Among these candidates, biogas is recently considered an emerging renewable energy that can be largely produced by a conventional process of anaerobic digestion of organic materials such as agricultural residues, animal manure, wastewater sludge, and organic fraction of municipal solid waste (MSW) including industrial wastes. Global production of biogas in Europe, China, and USA accounts for 90%, while about half of the remaining derives from Asia, such as Thailand and India^1^. In Thailand, biogas is produced from starch factories, biofuel industries, and livestock farms^1^. During 2019–2023, India planned to launch 5000 new plants of compressed biomethane gas (CBG)^1^. Although the current consumption of biogas is a small portion, there is a high potential for transforming overall energy system according to international energy agency (IEA)^1^. In addition, the World Biogas Association (2019) reported that the utilization of biogas could reduce around 10–13% of the world’s current greenhouse gas (GHG) emission^2^. In 2018, almost 60% of biogas was utilized for electricity generation and heat supply. However, upgradation of methane in the biogas into biomethane could be an important technological pathway of global growth^1^. Biogas primarily contains 50–70%v/v of methane (CH_4_), 30–50%v/v of carbon dioxide (CO_2_), and impurities such as nitrogen (N_2_), oxygen (O_2_), hydrogen sulfide (H_2_S), and humidity^3^. There are several existing technologies for upgradation to produce biomethane by CO_2_ separation such as water scrubbing, pressure swing adsorption, and chemical treatment^3^. By these traditional methods, the CO_2_ is discarded and only biomethane is supplied to the gas networks^4^. In general, the separation processes are costly as high pressure or additional chemicals are required^3^. Recently, it has been recognized that hydrogenation of CO_2_ in the biogas to produce biomethane would be a promising way for full utilization of biogas with benefits for renewable energy storage and abatement of CO_2_ emission. Several commercial plants that produce biomethane to substitute to natural gas are currently located in Denmark, Sweden, Germany, and The Netherlands^4^.

The hydrogenation of CO_2_ (Eq. 1) over heterogeneous catalysts is newly proposed for effective conversion of CO_2_ in the biogas into biomethane without additional costly separation processes^5^. In this regard, the hydrogenation coupling with hydrogen (H_2_) from electrolysis could be a potential pathway for seasonal storage of renewable energy and provide full utilization of biogas with great benefits on reducing GHG emission^4^. Power to gas (PtG) is a technology for the conversion of renewable electricity from solar energy to hydrogen gas by electrolysis^4^. However, hydrogen is not an ideal energy storage as it requires high compression (> 100 bar) to obtain only moderate density of energy^4^. Meanwhile, methane is considered a common energy carrier^4^. Methane has three-fold energy density with respect to hydrogen, while the facilities and infrastructure for transport and storage of methane are well-established^4^. Storing hydrogen in a form of methane, also known as power to methane (PtM) technology, can be achieved by hydrogenation of CO_2_ or Sabatier reaction^6^. Currently, electrolysis and hydrogenation of CO_2_ have shown high efficiencies of 65–85% and 77–83%, respectively^6^.

1 $${\text{CO}}_{2} + {\text{ 4H}}_{2} \to {\text{CH}}_{4} +{\text{ 2H}}_{2} {\text{O}}\quad \Delta {\text{H}}_{{{\text{298K}}}} {{ = }} - {\text{165 kJ}}\; {\text{mol}}^{{ - 1}}$$

Shildhauer et al.^7^ reported that the stable operation of the hydrogenation of CO_2_ in raw biogas could be demonstrated in  a bubbling fluidized bed reactor for 1100 h. Dannesboe et al.^4^ showed the hydrogenation of CO_2_ in biogas in a full-scale reactor for 1000 h without complications. The biomethane of 96% yield was produced with relative stable operation and slow deactivation by sulfur compounds. The sub-stoichiometric ratio of 3.9 was found to be optimal, preventing carbon deposition. Gaikwad et al.^8^ integrated processes of desulfurization, hydrogenation of CO_2_ and electrolysis for demonstration of biogas plant in Denmark. From above examples, although the upgradation of methane in the biogas could be ready for implementation at industrial scale, there are several issues to be concerned such as heat management and hotspots, carbon deposition, expensive H_2_, and requirement of higher CH_4_ purity. The hydrogenation of CO_2_ is a highly exothermic reaction. The accumulation of intensive heat would cause hotspots inside the reactor and potentially lead to thermal sintering of metal catalyst, or even thermal runaway and explosion^9^. The distinct hotspot formations can strongly influence the catalyst durability and the process safety^10^. Among promising metal catalysts, nickel (Ni) has acceptable high activity (Ru > Fe > Ni > Co > Mo) with the highest selectivity for CH_4_ formation (Ni > Co > Fe > Ru)^11^. In addition, its availability and economical prices make it widely used in industries. Nickel can be supported on various metal oxides (i.e., Al_2_O_3_, SiO_2_, etc.) to increase its dispersion. Recently, it is reported that nickel supported on cerium oxide (CeO_2_) exhibits an excellent performance catalyst for hydrogenation of CO_2_ into CH_4_^12,13^. The reversible valence change (Ce^4+^ and Ce^3+^) of CeO_2_ with high oxygen transport capacity provides unique properties such as high oxygen lattice concentration, high oxygen vacancies, and basic surface for CO_2_ adsorption that are useful for catalysis applications^14^. It recently reported that CeO_2_ supported with Ni catalysts exhibits a superior activity for hydrogenation of CO_2_ than other metal oxides^15^. However, CeO_2_ materials which are rare earth metal oxide, are expensive and low surface area materials. The high surface area support materials [mesoporous silica, metal organic framework (MOF), carbon, etc.,] with low prices would be preferred. The MOF structure is limited to low temperature reaction, while carbon supports show low performance for hydrogenation of CO_2_. Recently, mesoporous silica such as Mobil Composition of Matter No. 41 (MCM-41), containing aluminum (Al-MCM-41) could be prepared from abundant and cheap natural kaolin^16^. The use of Al-MCM-41 as catalyst support coupled with cerium promoter would be promising for hydrogenation of CO_2_^16^. In our previous work, the formula of Ni-Ce/Al-MCM-41 has been optimized for the hydrogenation of CO_2_^17^. However, the parameters including pressure, temperature, and feed flowrate are required for further fine-tuning to obtain the optimum condition.

In this study, we reported the experimental data from the upgradation of methane in the biogas by hydrogenation of CO_2_ in a prototype reactor with the comparison between single pass operation and double pass operation. The Ni-Ce/Al-MCM-41 optimized from the previous study was used as catalyst for search of optimum condition and stability test. The process conditions including temperature, feed flowrate, and pressure were investigated. The long-term run of upgradation process was operated in the vertical fixed bed reactor with double operation for 458 h to prove the durability of the catalyst and operational capability of the reactor. In addition, the comprehensive characterization of fresh and spent catalysts was performed to find the relationship between the catalyst properties and its catalytic performance.

## Experimental

### Catalyst preparation

The Ni-Ce/Al-MCM-41 catalyst was synthesized by hydrothermal described in our previous work^17^. First, catalyst support was prepared from kaolin by thermal treatment and acid leaching. Kaolin was heated at 650 °C for 2 h in a muffle furnace. After that, the sample was leached by 2.75 M H_2_SO_4_ at 90 °C for 2 h. The sample was washed and dried at 110 °C overnight. Then, 10 g leached kaolin was dispersed in aqueous solution (400 ml distilled water) of 1.1 g CTAB, 1.1 g PEG (4000), and 15 g NaOH, and stirred for 18 h. Then, Ni(NO_3_)_2_·6H_2_O and Ce(NO_3_)_3_·6H_2_O were added, stirred for another 1 h. The pH of solution was adjusted to 9 by HCl solution, and stirred for another 1.5 h. Then, the slurry was hydrothermally treated at 110 °C in Teflon-lined autoclave for 24 h. After that, the sample was washed and dried at 110 °C overnight. The obtained catalyst sample was calcined at 550 °C for 3 h (ramp 1 °C min^−1^). The optimized composition of Ni-Ce/Al-MCM-41 catalyst (reduced form) contains 10 wt%Ni and 50 wt%Ce supported on Al-MCM-41. The catalyst powder was compressed at 40 MPa for 15 min to form a tablet, and the tablet was cracked into small pieces of granules with particle size of 300–425 μm.

### Upgradation process

**Figure 1:**
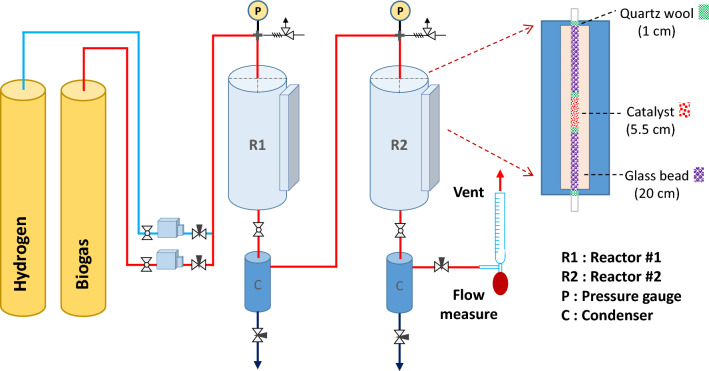
Schematic diagram of prototype reactor for upgradation process with double pass operation.

The upgradation of methane in biogas by hydrogenation of CO_2_ was performed in a prototype reactor with double pass operation as schematically shown in Fig. 1. The catalyst granules of each 5 g were packed in each reactor tube (∅ = 3/8 in. 70 cm height, SUS316, Swagelok) to obtain 10 g in total. The quartz wool (4 μm fiber, Ohio Valley Specialty) together with glass bead (∅ = 3 mm, Kemaus) was used for immobilization and packing of the catalyst bed inside the reactor. The reaction heat was supplied by an electric tube furnace of vertical arrangement (65 cm height with 51 cm effective heating zone, max. 1200 °C, 280VAC, 50 Hz, 15 A, 3.4 kW). The temperature was controlled by PID controller (Omron, EAN-H) and thermocouple (Type K, ∅ = 5 mm).

The temperature profile along the reactor tube was monitored by temperature indicators (HANYOUNG NUX, TP3) and thermocouples (Type K, ∅ = 3 mm) of 10 positions with distance interval of 2.5 cm. The pressure of the system was controlled by adjustable back pressure regulator (*GO*^*®*^ regulator, BP-3 Series, control range 0–51.8 bar, SUS316 diaphragm PTFE liner) installed at the outlet stream. The condenser (capacity 1 L) was equipped at the bottom of reactor tubes. The cooling bath (LAUDA^®^, Alpha RA24, max. 15 l min^−1^) was employed to circulate cooling water at 5 °C for condensing and trapping the water vapor. Prior to the reaction, the catalyst was activated by reduction with ultra-high purity H_2_ (UHP, 99.999%) at 100 ml min^−1^, 10 °C min^−1^ to 500 °C for 1 h, and then cooled down to the reaction temperature of 350 °C. Once the reactor temperature reached 350 °C, biogas (UHP gas mixture of 40%CO_2_ and 60%CH_4_) as model gas together with H_2_ at ratio of CO_2_:H_2_ = 1:4 was continually fed into the reactor. The product gas at downstream with dry basis was analyzed by Gas chromatograph (Shimadzu, GC-8A, column: i.d. 3 mm, 1.5 m length with active carbon, INJ/DET 120 °C, COL 100 °C, 100 mA, TCD) in every 10 min. The flow rates of the inlet gas were controlled by a thermal mass flow controller (Brooks^®^, SLA5800 Series, SLA5850/60, Viton) with meter (Brooks^®^, Model 0254 Series, four channel secondary electronics), while the flow rate of the outlet gas was measured by a soap bubble meter. After the reaction, the spent catalyst was collected for characterization.

### Product analysis

The catalytic activity of Ni-Ce/Al-MCM-41 catalyst and the upgradation efficiency of process were evaluated from CO_2_ conversion (X_CO2_) and selectivity (*S*_*i*_) of gas products (*i* = CH_4_ and CO) (Eqs. 2, 3).

2 $${\text{X}}_{{{\text{CO}}_{2} }} (\% ) = \frac{{[{\text{CO}}_{2} ]_{{{\text{in}}}} {\text{ }} \times {\text{ F}}_{{{\text{in }}}} -[{\text{CO}}_{2} ]_{{{\text{out}}}} \times {\text{ F}}_{{{\text{out}}}} }}{{[{\text{CO}}_{2} ]_{{{\text{in}}}} \times {\text{ F}}_{{{\text{in }}}} }} \times 100$$

3 $${\text{S}}_{{{\text{i }}}} (\% ) = \frac{{[{\text{CH}}_{4} ]_{{{\text{out}}}} \times {\text{F}}_{{{\text{out }}}} }}{{([{\text{CH}}_{4} ]_{{{\text{ out}}}} + [{\text{CO}}]_{{{\text{ out}}}} ) \times {\text{F}}_{{{\text{out}}}} }} \times 100$$

The flowrate of product gas, biomethane purity, and gas hourly weight velocity (GHSV) were calculated using Eqs. (4–6). *F*_*i*_ (ml min^−1^) is the flow rate of gas component *i*, where [*i*] represents the fraction of CH_4_, CO_2_, H_2_ or CO. *F*_in_ and *F*_out_ (ml min^−1^) are the total flow rate of inlet and outlet gas*. m*_*catalyst*_ is the weight of catalyst.

4 $${\text{Volumetric Flowrate, (F}}_{{\text{i}}} {\text{, ml}}{\mkern 1mu} \,{\text{min}}^{{ - 1}} ) = [{\text{i}}]_{{{\text{out}}}} \times {\text{F}}_{{{\text{out}}}}$$

5 $${\text{Purity}}\,{\mkern 1mu} {\text{of}}\,{\mkern 1mu} {\text{Biomethane}}\,{\mkern 1mu} (\% ) = \frac{{[{\text{CH}}_{{\text{4}}} ]_{{{\text{out}}}} \times {\text{F}}_{{{\text{out}}}} }}{{([{\text{CH}}_{{\text{4}}} ]_{{{\text{ out}}}} + [{\text{CO}}_{{\text{2}}} ]_{{{\text{ out}}}} + [{\text{H}}_{{\text{2}}} ]_{{{\text{ out}}}} + [{\text{CO}}]_{{{\text{ out}}}} ) \times {\text{F}}_{{{\text{out}}}} }} \times {\text{100}}$$

6 $${\text{Gas}}{\mkern 1mu} \,{\text{Hourly}}\,{\mkern 1mu} {\text{Space}}\,{\mkern 1mu} {\text{Velocity}}\,{\mkern 1mu} ({\text{GHSV}},{\text{ml}}\,{\text{g}}^{{ - 1{\mkern 1mu} }} \,{\text{h}}^{{ - 1}} ) = \frac{{{\text{F}}_{{{\text{in}}}} }}{{{\text{m }}_{{{\text{catalyst}}}} }}$$

### Catalyst characterization

The catalysts were characterized by various techniques to observe the changes in physicochemical properties of calcined, reduced, and spent catalysts. The XRD pattern was obtained by X-ray diffraction (XRD, Bruker, D2 Phaser) with 2θ = 10°–90°. The crystallite sizes of Ni and CeO_2_ (*d*_*Ni*_ and *d*_*Ce*_) were then estimated by Scherrer equation using full width at half maximum (FWHM) of prominent XRD peak. The morphology was captured by field emission scanning electron microscope (FE-SEM, JEOL, JSM-7610F) at operating voltage of 10 kV. The carbon deposition on the surface of catalysts was analyzed by thermogravimetric analysis (TGA, Mettler Toledo, TGA/DSC1) under O_2_ flow of 50 ml min^−1^ at 10 °C min^−1^ to 800 °C. The Raman spectra were recorded by Raman microscope (Horiba, XploRA PLUS) with wavelength at 532 nm. The textural properties were measured by N_2_ sorption measurement (Micromeritics, TriStar II 3020). The specific surface area (S_BET_), pore volume (*V*_*meso*_, *V*_*micro*_, *V*_*total*_), and pore size (*d*_*pore*_) are reported.

## Results and discussion

### Upgradation of methane in the biogas by hydrogenation of CO_2_

**Figure 2:**
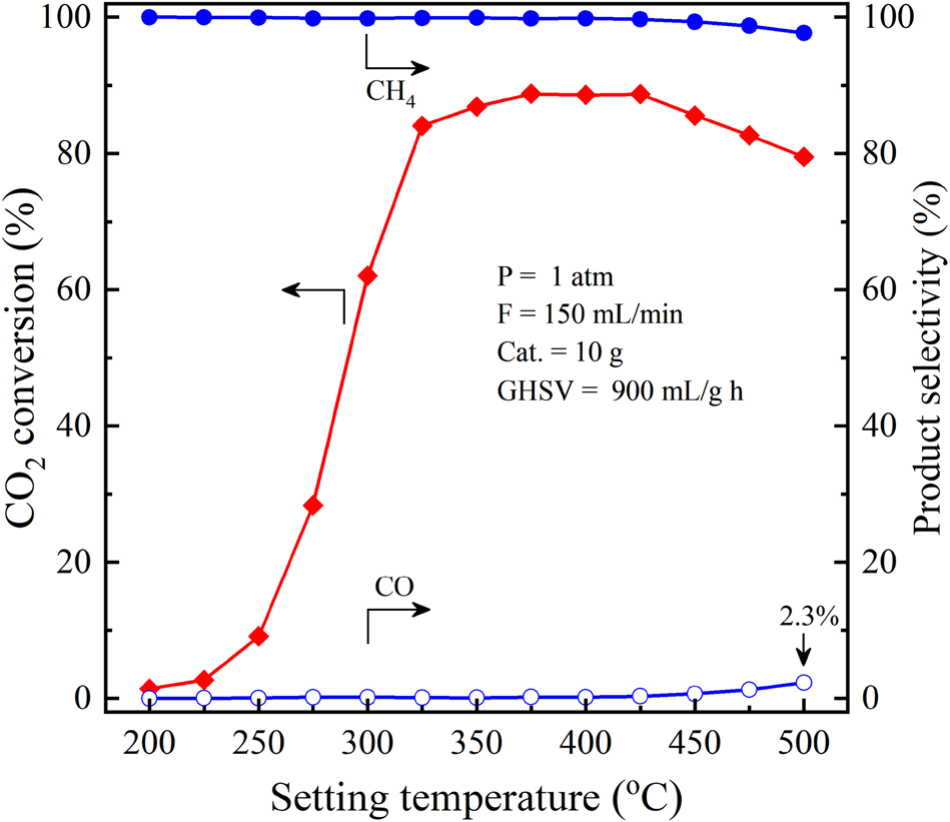
The effects of temperature on the upgradation performance (CO_2_ conversion and product selectivity) in the prototype reactor with double pass operation.

Upgradation of methane in the biogas by hydrogenation of CO_2_ (Eq. 1) was carried out. To maximize the CO_2_ conversion as well as CH_4_ production yield, the process was operated by using double pass operation. The reactor system consists of two reactor tubes connected in series (Fig. 1). Between the reactor tubes, there is a condenser for removal of water vapor from the outlet stream before dried gases enter the second reactor. The remained/unreacted CO_2_ and H_2_ from the first reactor can be further reacted in the second reactor due to the equilibrium shift, then producing more CH_4_. In other words, the reaction equilibrium was shifted to more producing the methane gas by double pass operation. The impact of double pass operation is explained in the next section. Herein, the parameters affecting the upgradation efficiency such as temperature, flowrate, and pressure were investigated. First, the effects of temperature on the efficiency of upgradation process are investigated. Figure 2 shows the CO_2_ conversion and the product selectivity including CH_4_ and CO gases at different temperatures^15^. It was found that the CO_2_ conversion greatly increased from 2 to 83% by elevating the temperature from 200 to 325 °C, showing the reaction kinetic is highly limited at low temperature. Meanwhile, the CO_2_ conversion can achieve the high value and remained unchanged at the temperature range of 325–450 °C. However, the temperature higher than 450 °C is not likely to be suitable for the upgradation process, as the CO_2_ conversion and CH_4_ production yield decreased with slight increase in the undesired CO gas products of 1.3% and 2.3% at 450 °C and 500 °C, respectively. At 200 °C, the CO selectivity is 0%, while it is in a range of 0.02–0.16% at 225–400 °C. The result was due to the exothermic nature of hydrogenation of CO_2_^13^. The equilibrium conversion of CO_2_ as well as CH_4_ selectivity decrease at high temperature^18^.

**Figure 3:**
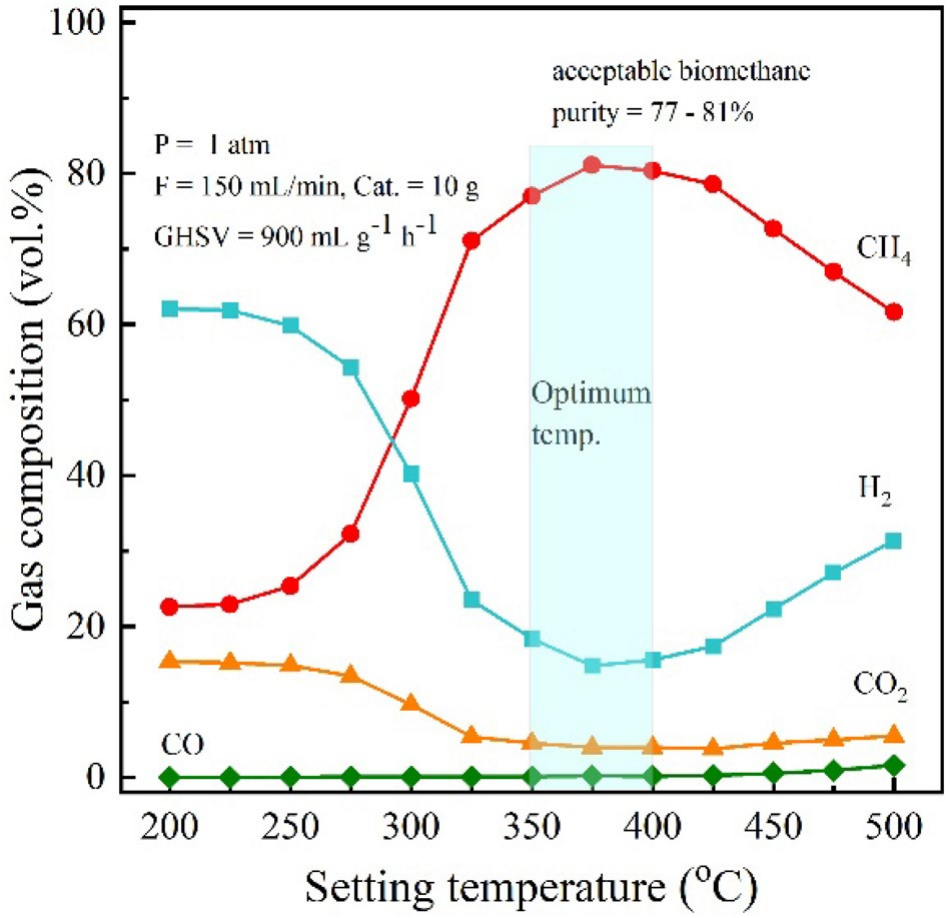
The composition of gas product after upgradation in the prototype reactor with double pass operation.

In terms of the efficiency of upgradation process, the purity of biomethane is considered. Figure 3 shows the final composition of outlet gas stream at different temperatures. The initial composition of input gas is 15%CO_2_, 23%CH_4_, and 62%H_2_. As the temperature increased, it was found that the fractions of CO_2_ and H_2_ decreased, while CH_4_ fraction increased, attributed to hydrogenation of CO_2_ to CH_4_. In the temperature range of 350–400 °C, the process can provide not only the high CO_2_ conversion > 80% with high CH_4_ selectivity *ca.* 100% (Fig. 2), but also the contents of methane are high at 77–81% (Fig. 3). In addition, the temperature range of 350–400 °C provided only small CO content less than 0.20%. More specifically, low temperature range of 200–325 °C produced the extremely low CO content of 0.00–0.10%, while high temperature range of 425–500 °C produced slightly higher CO content of 0.26–1.57%. Consequently, the suitable operation temperature was chosen at moderate temperature of 350 °C, and then for the further tests.

**Figure 4:**
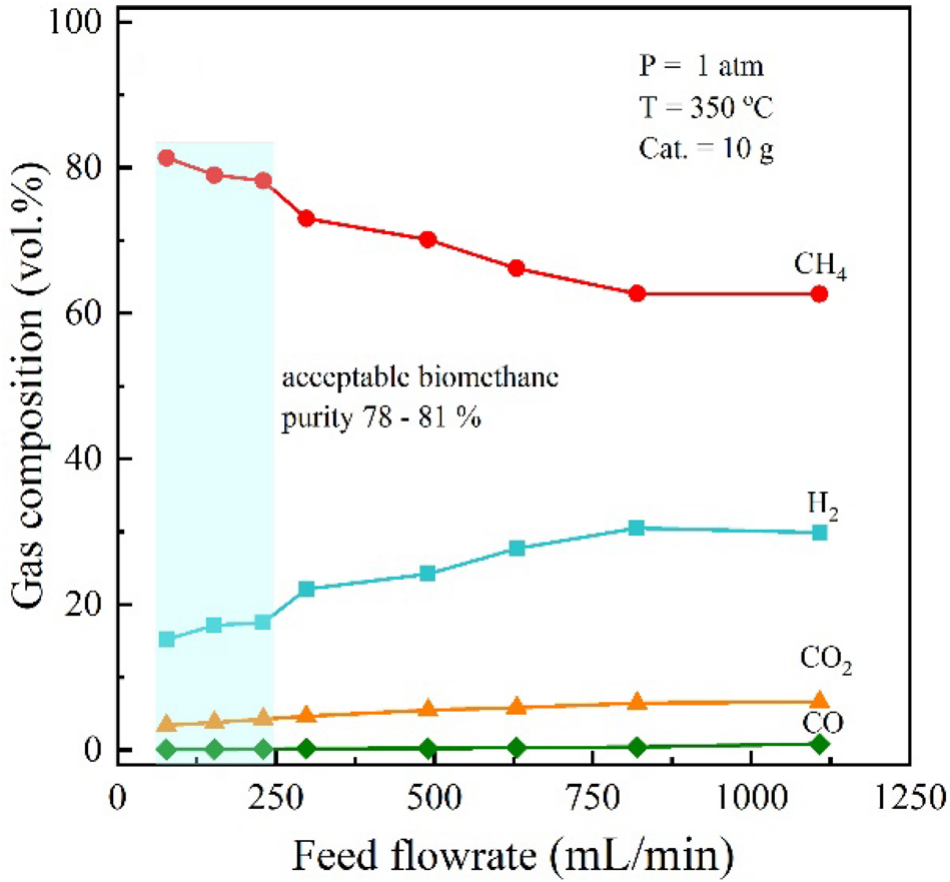
The effects of flowrate of feed on the upgradation in terms of gas compositions in the prototype reactor with double pass operation.

Next, the effects of flowrate and pressure on the efficiency of upgradation process are investigated. The flowrate of biogas varied from 77 to 1108 ml min^−1^ over Ni-Ce/MCM-41 catalyst with total weight of 10 g, corresponding to GHSV of 462–6720 ml g^−1^ h^−1^. It should be noted that the tests intentionally employed the pure biogas without any dilution or carrier gas to simulate the upgradation process for actual implementation. Figure 4 shows the composition of product gas stream at different flowrates of biogas. In general, an increase in the flowrate of biogas would reduce residence time of the reactant gas passing through the catalyst bed. As a result, the CH_4_ production yield decreased, while fractions of H_2_, CO_2_, and CO increased with the increased flowrate. In addition, the CO content in the upgraded biogas was increased from 0.05 to 0.83% with increased flowrate from 77 to 1108 ml min^−1^ at 350 °C. Considering the effects of biogas flowrate in the investigated range, biomethane with purity of 78–81% was obtained at the biogas flowrate range of 77–250 ml min^−1^ as highlighted by light blue background. According to the announcement on NGV quality specifications from the Department of Energy Business, Ministry of Energy, Thailand^19,20^, the acceptable methane content would be higher than 65% for general vehicles, and more than 75% for special vehicles. Again, the flowrate of 150 ml min^−1^ was chosen as a representative of suitable condition of biogas flowrate for the further experiments.

**Figure 5:**
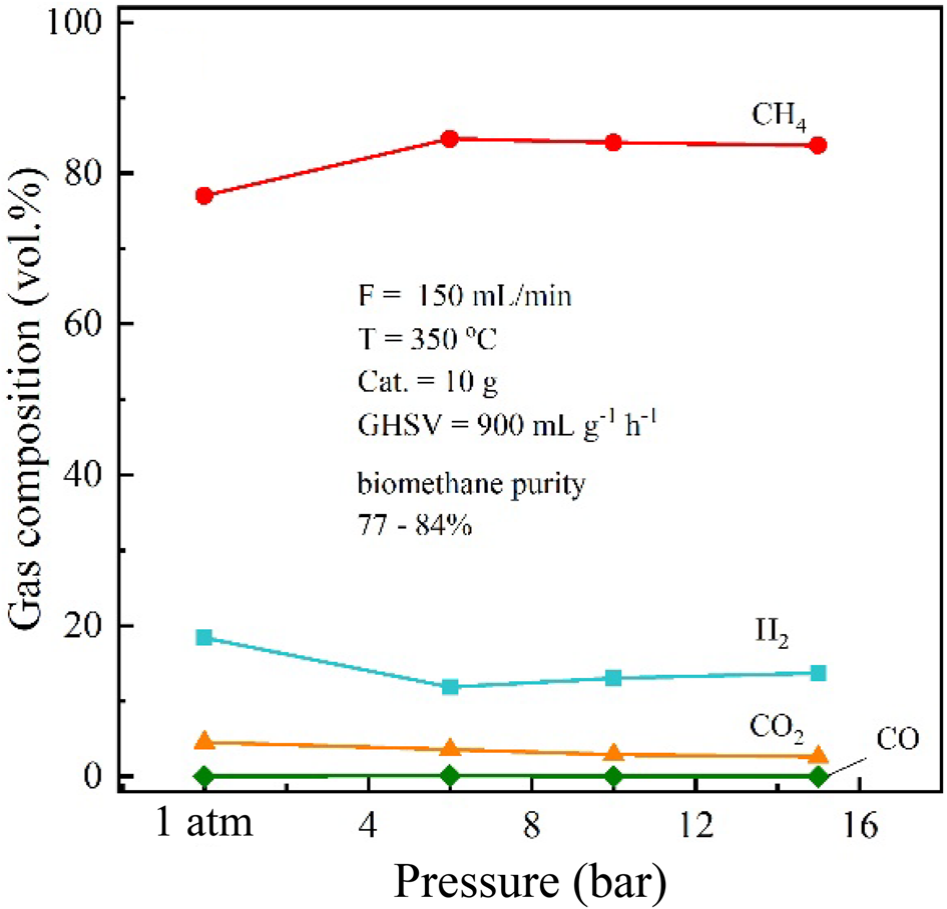
The effects of pressure on the upgradation in the prototype reactor with double pass operation.

Finally, the effects of pressure were investigated. It is well known that increasing pressure of gaseous reaction system would promote the reaction kinetic as well as increases the yield of products, especially the reaction that finally result in smaller total moles such as CO_2_ hydrogenation (CO_2_ + 4H_2_ → CH_4_ + 2H_2_O). Figure 5 shows the effects of pressure on the upgradation process. Considering the composition of biomethane products, the methane fraction increased from 78 to 83% by increasing the pressure from atmospheric pressure to 6 bar. This result is in line with the results reported previously^4^. However, a further increase in pressure from 6 to 15 bar shows unchanged in the CH_4_ yield. At this point, it should be noted that although the increased pressure shows a positive effect on the CH_4_ production yield, the improvement is very small. In addition, the CO content in the upgraded biogas was slightly decreased from 0.08 to 0.01% with the increased pressure from 1 atm to 15 bar at 350 °C, and 150 ml min^−1^.

**Figure 6:**
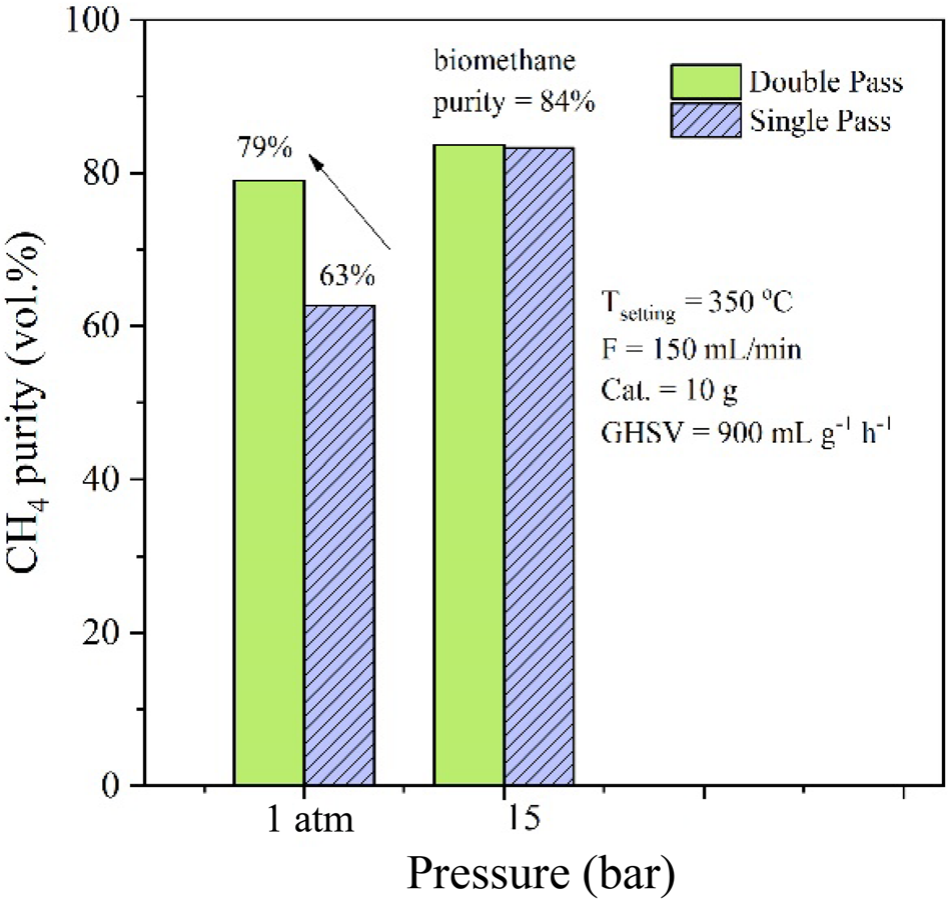
The comparison of methane purity from single pass operation/double pass operation at different pressures for upgradation of biogas in the prototype reactor.

**Figure 7:**
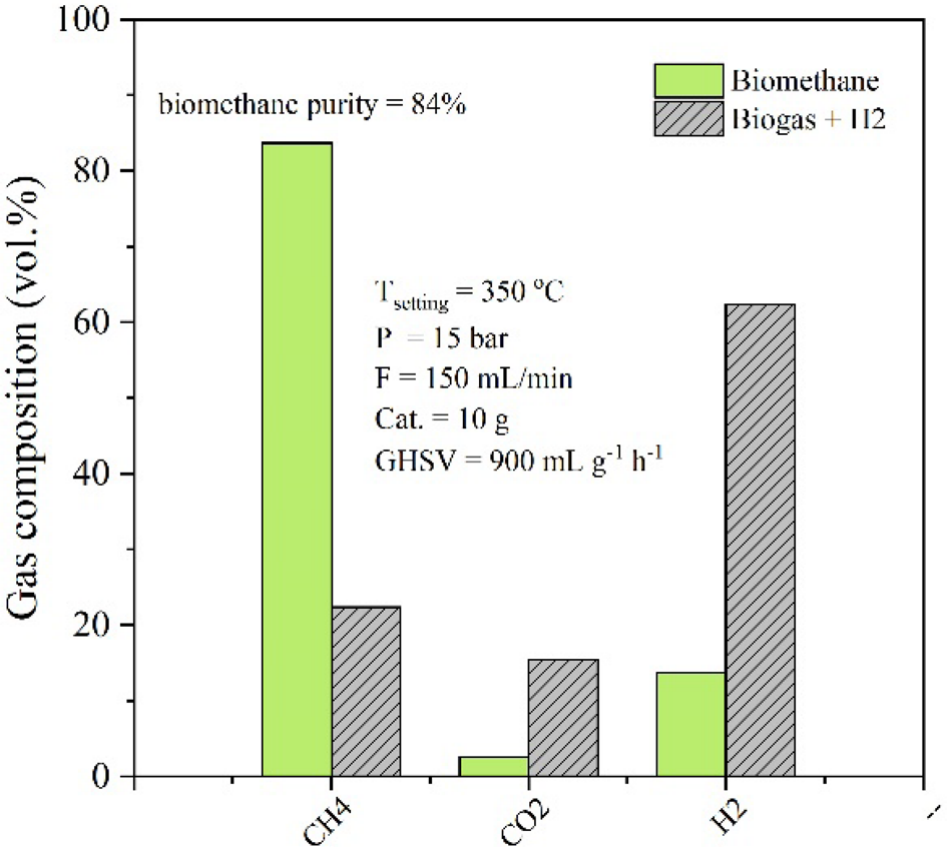
The comparison of the input flow composition and the output flow composition under the best condition for upgradation by double pass operation in the prototype reactor.

In this work, the reaction system is designed to possess two consecutive reactors for double pass operation. The double pass operation would remove the water vapor in the outlet gas from the first reactor, then the dried gas was introduced to the second reaction. Then a further reaction is proceeded, resulting in higher CH_4_ production. Dannesboe et. al reported the design of the double pass strategy could ensure a methane content/Wobbe index within the specifications of natural gas. The methane content was increased by the double pass design of the reactor. In addition, the experimental results showed that the reactor could be operated at full scale for 1000 h without complications^4^. The advantages of double pass operation were examined with comparison to the single pass operation as shown in Fig. 6. At the atmospheric pressure, the double pass operation can improve the methane fraction from 63 to 79% (∼15% improvement of biomethane purity). However, the double pass operation shows less important when the system was pressurized at 15 bar. The purity of biomethane is approximately the same at 84 vol% for both single and double pass operations at 15 bar. Figure 7 shows the comparison of the input flow composition and the output flow composition. The upgradation process was carried out at 350 °C, 15 bar, and GHSV of 900 ml g^−1^ h^−1^. The model biogas comprised of 40%CO_2_ and 60%CH_4_ was mixed with H_2_ at CO_2_/H_2_ ratio of 1/4, resulting in the input flow composition of 15%CO_2_, 23%CH_4_, 62%H_2_. It was found that the biogas was improved to products containing high CH_4_ content. The output flow composition consists of 84%CH_4_, 14%H_2_, and 2%CO_2_ as shown in Fig. 7. By several tests, this condition shows the best operation condition for upgradation of methane in the biogas by hydrogenation of CO_2_. The contents of CH_4_ and CO_2_ are comparable with biomethane obtained from the process by separation of CO_2_^21^. In addition, the concentrations of CH_4_ and CO_2_ in the product gas would meet the standard composition of biomethane^21^.

**Figure 8:**
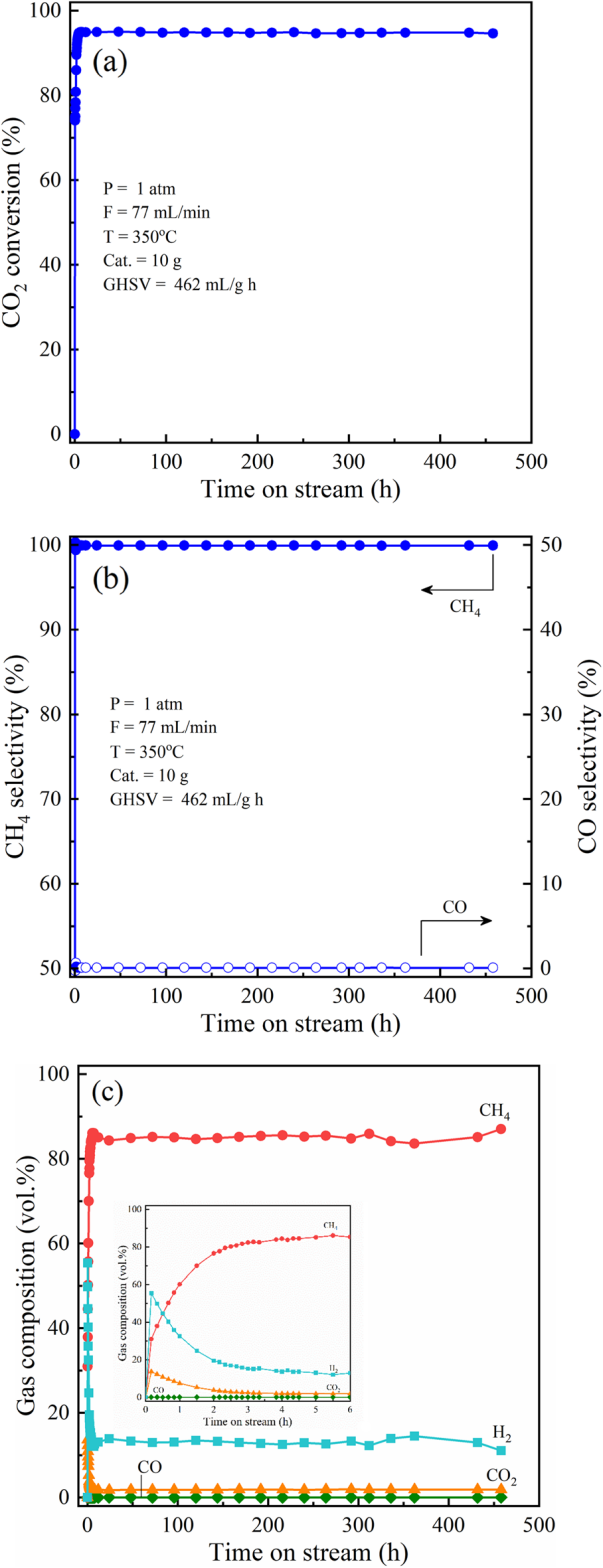
The catalytic performance during the stability test for 458 h: (**a**) CO_2_ conversion, (**b**) product selectivity, (**c**) gas compositions.

The long-time test of catalyst was carried out. Surprisingly, the catalyst shows an excellent catalytic stability for 458 h time-on-stream. In Fig. 8a, the CO_2_ conversion attained 94% and kept constant over the time on stream of 458 h. In addition, desired product selectivity achieved 100%, and there was unchanged as shown in Fig. 8b. The gas composition of outlet stream was analyzed as shown in Fig. 8c. The comprehensive characterization of fresh and spent catalysts was performed to relate the physicochemical properties of catalyst with its catalytic stability.

### Characterization of fresh and spent catalysts

**Figure 9:**
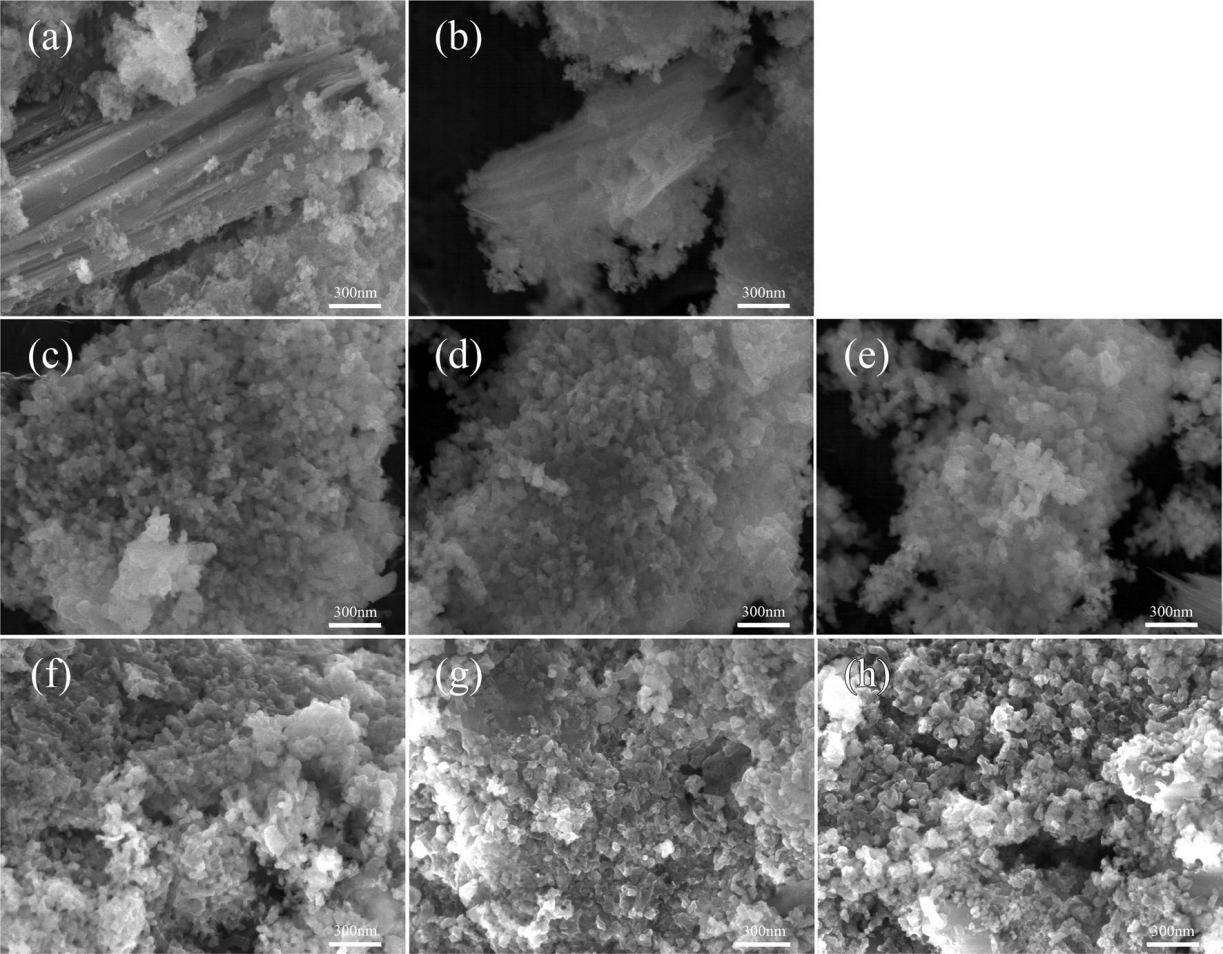
SEM images: (**a**) calcined, (**b**) reduced, (**c**) spent catalyst SR1_Top, (**d**) SR1_Middle, (**e**) SR1_Bottom, (**f**) SR2_Top, (**g**) SR2_Middle, and (**h**) SR2_Bottom.

The characterization of catalysts by various techniques was performed to determine the physicochemical properties of fresh and spent catalysts. The fresh catalysts consist of the calcined catalyst and the reduced catalyst. Meanwhile, the spent catalysts are obtained after the reaction with stability test for 458 h, collected from two reactors (SR1 and SR2) at three positions including top, middle, and bottom of catalyst bed (total bed lenght of 5.5 cm). Figure 9 shows SEM images of fresh and spent catalysts, illustrating the shape and surface morphology. In Fig. 9a, the particles with plate-like shape would be Al-MCM-41 as catalyst support, while the finer pseudo-spherical particles with spongy structure would be cerium oxide nanoparticles. The nickel particles could not be distinguished by SEM observation. In Fig. 9b, the reduced catalyst seems to have a similar morphology to the calcined catalyst, implying less effects from H_2_ activation on the catalyst morphology. However, the catalyst morphology was changed after the test for 458 h as shown in Fig. 9c–h. In the reactor #1 (SR1), the CeO_2_ particles increased in grain size after the stability test.

**Figure 10:**
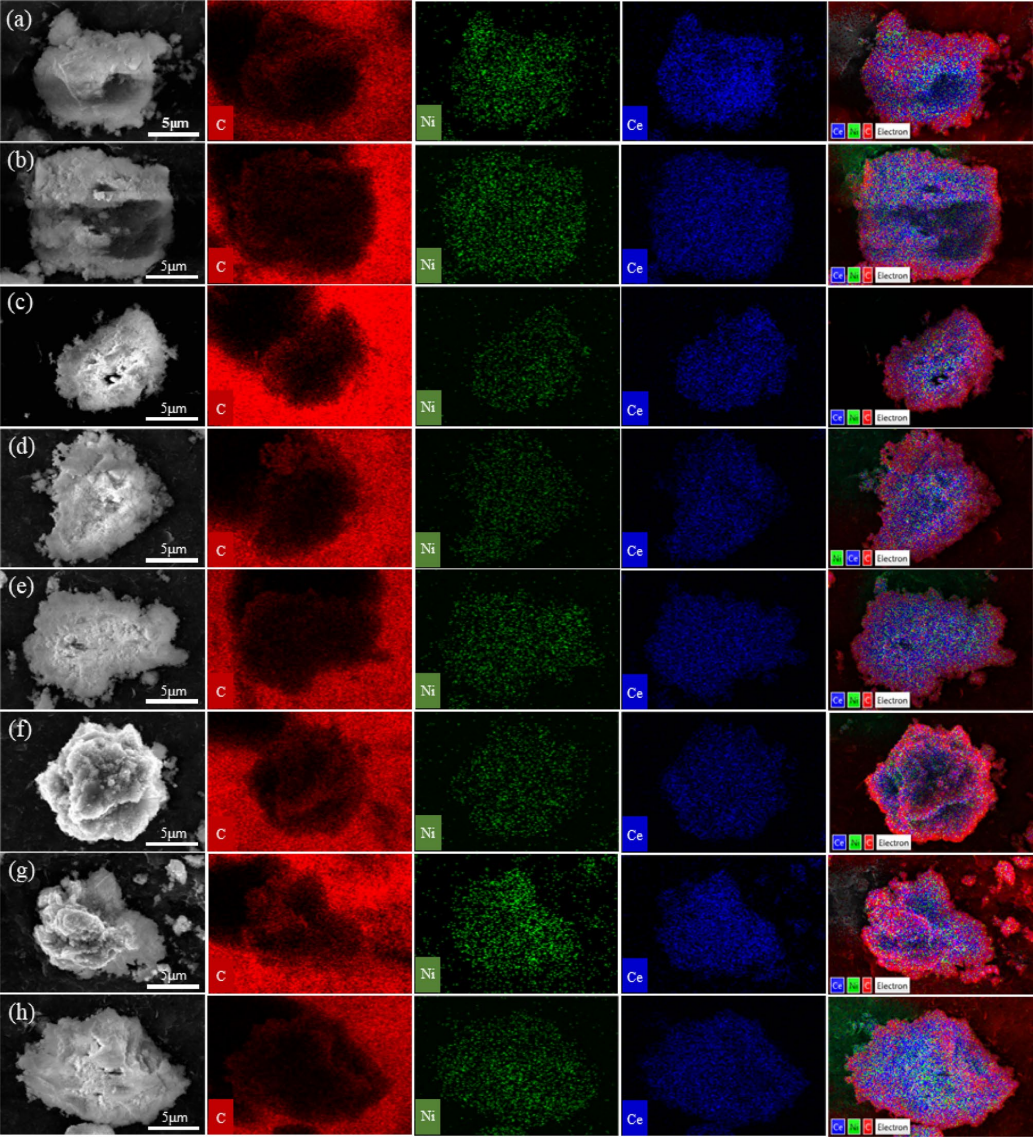
SEM images and its corresponding elemental mappings: (**a**) calcined, (**b**) reduced, (**c**) spent catalyst SR1_Top, (**d**) SR1_Middle, (**e**) SR1_Bottom, (**f**) SR2_Top, (**g**) SR2_Middle, and (**h**) SR2_Bottom.

Figure 10 shows the SEM images and their corresponding elemental mappings of fresh and spent catalysts. The dispersion of elements including carbon (C), nickel (Ni), and cerium (Ce) are mapped into red, green, and blue colors, respectively. The overlay of three components is also illustrated. It was found that the particle size of catalysts as well as surface morphology are indifferent as seen in the SEM images. In addition, the major components of catalyst (Ni and Ce) remain well dispersed after the long-term run for 458 h. On the observation of spent catalysts by the elemental mapping, it seems there is no significant agglomeration of catalyst metals after the reaction. However, the crystallite size of Ni calculated from the XRD data (Fig. 12) presented the larger particles observed. The Ni crystallite of reduced catalyst is 11.2 nm. After the reaction for 458 h, the Ni crystallite sizes of spent catalysts in the reactor #1, are 21.9, 17.9, and 14.2 nm for top, middle, and bottom, respectively. The accumulation of heat generated from exothermic reaction would result in the formation of the high temperature area or so-called hotspots, causing thermal sintering of catalyst metals^22^. The mechanism of thermal sintering involves surface diffusion or mobility of larger particles^23^. Thermal sintering could be the main influence for decreasing nickel surface area during hydrogenation of CO_2_^23^. This could be related to the Ni particles becoming the largest size of 21.9 nm, caused by metal sintering. Meanwhile, the Ni crystallites of spent catalyst collected from top, middle, and bottom of the reactor #2 are 14.5, 16.0, and 14.0 nm, respectively. Notably, it was smaller than those of the reactor #1. Interestingly, carbon deposition by CH_4_ cracking (endothermic reaction) observed by TGA is high at the front part (top), while the hot spot (exothermic hydrogenation of CO_2_) is shifted to the middle part of catalyst bed. The product gas from the reactor #1 with high concentration of CH_4_ would induce the cracking of CH_4_ in the reactor #2, even at low temperature of 350 °C. Consequently, the carbon species were more observed in the spent catalyst from the reactor #2 as seen in the overlay images (Fig. 10f–h). The carbon deposition might happen on the surface of catalyst by decomposition of methane (Eq. 7). Further characterizations of carbon deposition by TGA and Raman were performed, and the results are discussed in the next section.

7 $${\text{CH}}_{4} {\to} {\text{C}} + {\text{H}}_{2} \quad \Delta {\text{H}}_{{298{\text{K}}}} = + 74.9{\text{ kJ}}\,{\text{mol}}^{{ - 1}}$$

**Figure 11:**
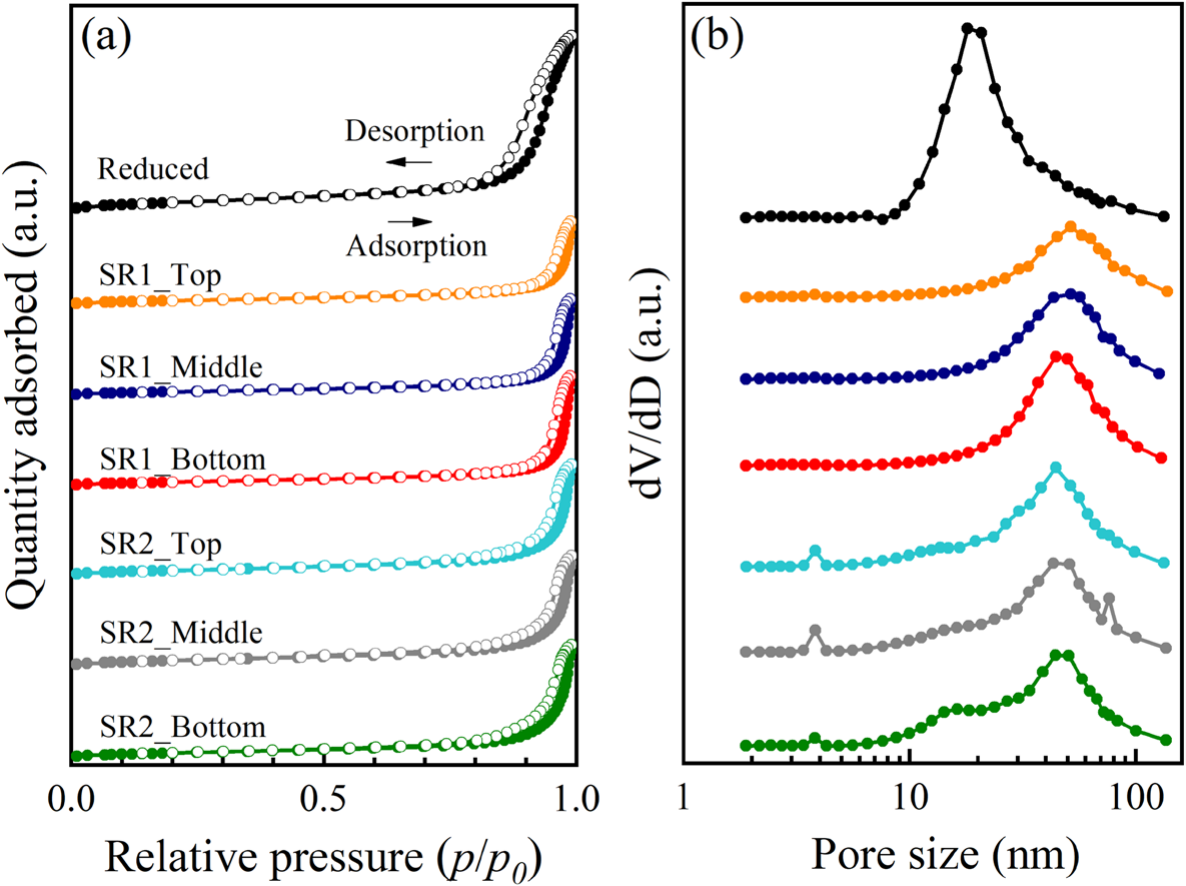
(**a**) N_2_ adsorption–desorption isotherms and (**b**) corresponding BJH pore size distributions.

After the reaction for 458 h, the spent catalysts collected from different positions of the reactors were analyzed by N_2_ sorption measurement. The N_2_ adsorption–desorption isotherms and BJH pore size distributions are presented in Fig. 11a,b, while the textural properties are listed in Table 1. In Fig. 11a, it is noticed that all isotherms contain a hysteresis loop with a knee at point B, representing a type IV(a) isotherm according to IUPAC classification. Although the knee at point B is relatively weak as the isotherms are plotted stacking, a gradual curvature of point B was present, indicating an overlap of monolayer coverage and the onset of multilayer adsorption^24^. In general, a type IV isotherm indicates that a material contains mainly mesopore structure of a pore size range of 2–50 nm. However, these isotherms also exhibit a large adsorption volume at a high relative pressure (*p*/*p*_*0*_ = 0.8–1.0), attributed to a presence of macropore structure (*d*_*pore*_ > 50 nm)^25^. This combined structure of mesopore and macropore is related to a broad pore size distribution shown in Fig. 11b. The hysteresis starting at a relatively high relative pressure (*p*/*p*_*0*_ > 0.5) is an indication of delayed condensation on the adsorption branch. In addition, the hysteresis exhibiting a steep and narrow loop represents a H1 type hysteresis loop^24^. The materials with type H1 loop would have uniform mesopores, such as mesoporous silicas (e.g., MCM-41, MCM-48, SBA-15, etc.)^24,26^. This confirms the existence of Al-MCM-41 and contribution of its mesoporous structure.

**Table 1:** Textural properties of fresh and spent catalysts collected from different positions in the reactors.

Sample	*S*_*BET*_ (m^2^ g^−1^)	*V*_*micro*_ (cm^3^ g^−1^)	*V*_*meso*_ (cm^3^ g^−1^)	*V*_*total*_ (cm^3^ g^−1^)	*d*_*meso*_ (nm)
Fresh catalyst_Reduced	34.8	0.0011	0.1852	0.1863	21.1
Spent catalyst_SR1_Top	17.3	0.0010	0.0866	0.0876	25.0
Spent catalyst_SR1_Middle	17.0	0.0008	0.1004	0.1013	28.3
Spent catalyst_SR1_Bottom	16.9	0.0008	0.1148	0.1156	31.9
Spent catalyst_SR2_Top	23.0	0.0016	0.1171	0.1187	22.8
Spent catalyst_SR2_Middle	25.0	0.0019	0.1152	0.1171	22.4
Spent catalyst_SR2_Bottom	20.5	0.0012	0.1182	0.1194	26.3

*V*_*micro*_ micropore volume by t-plot method, *d*_*meso*_ average mesopore diameter by BJH method.

A decrease of (I) adsorption volume of hysteresis of all spent catalysts (Fig. 11a) and (II) derivative pore volume (*dV*/*dD*) in Fig. 11b would indicate the reduction of catalyst porosity after the reaction. In general, the reduction of catalyst porosity during the reaction can be caused by several deactivation mechanisms such as a collapse/fusion of pore structure, metal sintering, pore blocking, etc. However, it was found that the position of hysteresis was shifted to higher relative pressure (Fig. 11a), while peaks of pore size were shifted to a larger pore size range (Fig. 11b). These results imply the reduction of catalyst porosity was caused by a collapse/fusion of pore structure, as the pore blocking usually reduces surface area with smaller pore size.

Quantitative data of catalyst porosity including specific BET surface area (*S*_*BET*_), pore volumes (*V*_*micro*_, *V*_*meso*_, *V*_*total*_), and average mesopore size (*d*_*meso*_) are summarized in Table 1. It was found that the *S*_*BET*_ values are in a range of 17.0–34.8 m^2^ g^−1^, representing a material having low-to-moderate surface area. The amount of micropore volume (*V*_*micro*_ = 0.0008–0.0019 cm^3^ g^−1^) is negligible, less than 0.2% compared to mesopore volume (*V*_*meso*_ = 0.1004–0.1852 cm^3^ g^−1^). The porosity of catalysts mainly consists of mesopore. Consequently, the pore size is presented as the average mesopore by BJH method.

In Table 1, the reduction of porosity after reaction can be quantitatively compared. The porosity of initial reduced Ni-Ce/Al-MCM41 catalyst is first recognized as *S*_*BET*_ = 34.8 m^2^ g^−1^, *V*_*micro*_ = 0.0011 cm^3^ g^−1^, *V*_*meso*_ = 0.1852 cm^3^ g^−1^, *V*_*total*_ = 0.1863 cm^3^ g^−1^, and *d*_*meso*_ = 21.1 nm. After the reaction for 458 h, it was found that the *S*_*BET*_ and *V*_*meso*_ of spent catalysts in the reactor #1 decreased to 16.9–17.3 m^2^ g^−1^ and 0.0866–0.1148 cm^3^ g^−1^. The hydrogenation of CO_2_ would generate highly exothermic heat, causing the collapse of pore structure. On the other hand, the spent catalysts in the reactor #2 that involves the milder reaction activity could remain the higher *S*_*BET*_ and *V*_*meso*_ values. Interestingly, the carbon deposits in the reactor #2 would have less contribution in decreasing the *S*_*BET*_ and *V*_*meso*_ of catalysts.

**Figure 12:**
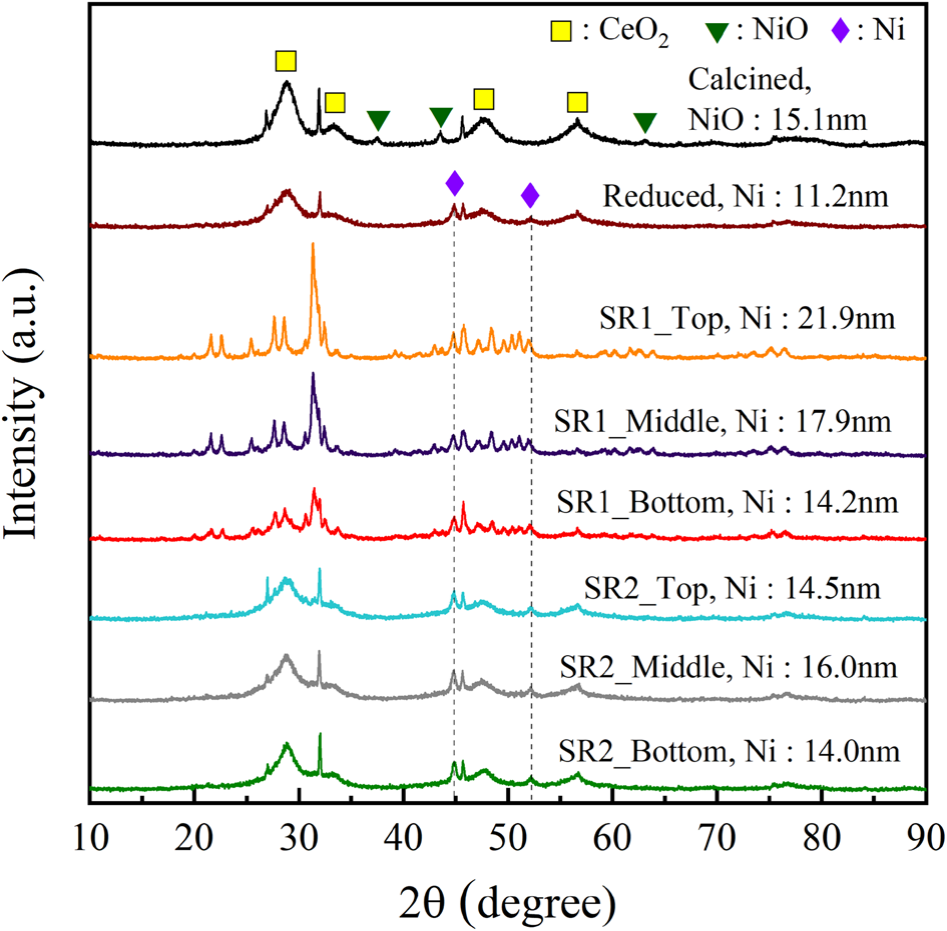
XRD patterns of fresh and spent catalysts from different positions of catalyst bed after the stability test for 458 h.

Figure 12 shows XRD patterns of fresh and spent catalysts. The presence of NiO in the calcined catalyst was confirmed by the peaks at 37.2°, 43.1°, and 63.2°, assigned to the diffraction of [111], [200 and [220] planes (JCPDS Card No. #47-1049), respectively^13^. The reduced catalyst shows the presence of Ni observed by the peaks at 44.3° and 51.8° assigned to the [111] and [200] planes (JCPDS 65-0380), respectively^13^. The highly crystalline CeO_2_ was determined by the peaks at 28.7°, 33.2°, 47.8°, and 56.2°, assigned to the [111], [200], [220], and [311] planes (JCPDS 43-1002), respectively^13^. The impurity such as quartz in kaolin was observed at the peak at 26.6°^16^. It was observed that the structural properties of spent catalysts from the reactor #1 are clearly different from those of the reactor #2. This would confirm the effects of hotspots in the reactor #1. The fresh catalysts including calcined and reduced contain the nickel crystallite sizes of 15.1 nm and 11.2 nm for NiO (43.5°) and Ni (44.8°), respectively^15^. After the reaction, the Ni crystallite sizes of spent catalysts collected from top, middle, and bottom of the reactor #1 became larger in sizes of 21.9, 17.9, and 14.2 nm, respectively. The largest Ni size of 21.9 nm is located at the top of catalyst bed, attributed to the intensive heat accumulation or hotspots. Meanwhile, the Ni sizes of those from the reactor #2 are remained small at 14.5, 16.0, and 14.0 nm, respectively. In the reactor #2, the higher methane fraction would promote a dissociation of CH_4_ (methane cracking), resulting in carbon deposition. Since the cracking of CH_4_ is an endothermic reaction, the hotspots are suppressed, resulting in the lesser Ni sintering, especially at the top of catalyst bed. Further characterization of fresh and spent catalysts by TGA and Raman was carried out to determine the carbon deposition.

**Figure 13:**
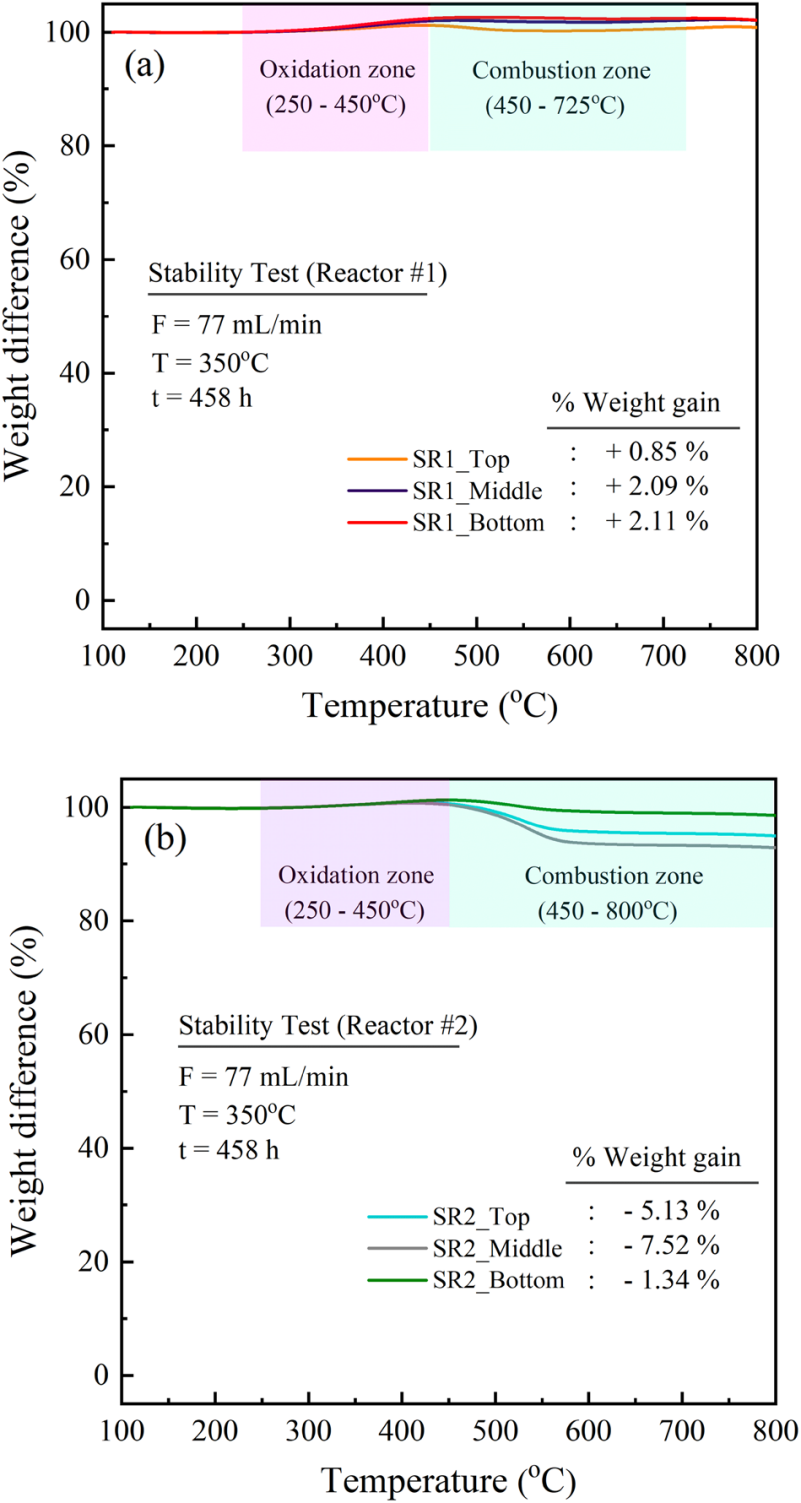
TGA curves of spent catalysts collected from different positions of catalyst bed after the test run for 458 h: (**a**) reactor #1, and (**b**) reactor #2.

**Figure 14:**
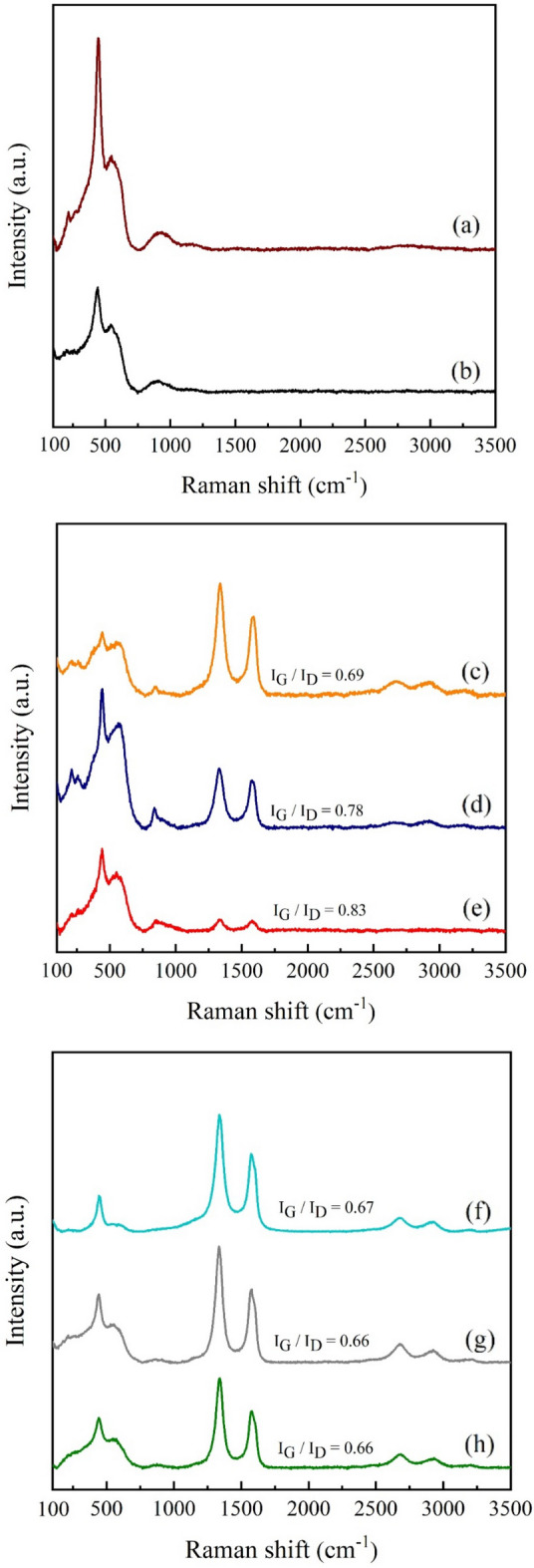
Raman spectra of Ni-50Ce/MCM-41 catalysts: (**a**) calcined, (**b**) reduced, (**c**) SR1_Top, (**d**) SR1_Middle, (**e**) SR1_Bottom, (**f**) SR2_Top, (**g**) SR2_Middle, and (**h**) SR2_Bottom.

Figure 13 shows the TGA curves of spent catalysts collected from different positions of catalyst bed after the stability test run for 458 h. The sample in TGA analysis was heated under the flow of oxygen gas. Therefore, the weight loss of sample (< 100%) is typically considered the combustion of carbon deposits, while the weight gain of samples (> 100%) would be caused by the oxidation of Ni metals. In Fig. 13a, it was found that the spent catalysts from the reactor #1 (SR1) gained the weight at the temperature range of 280–540 °C, representing the oxidation zone of Ni, while a slight decrease in weight of sample was observed at 540–725 °C, indicating the combustion zone. Since the amount of oxidation phase is larger than those of combustion component, the final weight of samples is more than 100%. In addition, the final weight gain of samples shows an increasing trend of 0.85, 2.09, and 2.11% for the samples collected from top, middle, and bottom position, respectively, corresponding to the different amount of carbon deposition as confirmed by Raman analyses (Fig. 14). Figure 13b shows the TGA profiles of samples collected from the reactor #2. It was found that weight loss was observed in all samples. The carbon deposition was clearly observed in the reactor #2, caused by methane decomposition.

After the stability test, the presence of carbon deposits on the surface of spent catalysts was analyzed by Raman spectroscopy. This technique can analyze even a small amount of carbon species on the catalyst surface. Figure 14 shows a comparison of Raman spectra plot of fresh and spent catalysts. In general, the nickel species such as Ni and NiO are rarely observed by Raman analysis, while a distinct peak at 468 cm^−1^ is assigned to the presence of crystalline CeO_2_^27^. The bands at 1300–1400 cm^−1^ and 1500–1600 cm^−1^ indicated D band and G band of carbon structure, respectively. The G band indicated the graphitic carbon, while the D band represents the disordered carbon^28^. There are no G and D bands observed on the calcined and reduced catalysts (a–b), indicating no carbon species in the fresh catalysts. On the other hand, G and D bands were observed for all samples of spent catalysts. In the reactor #1, the difference in the intensities of G and D bands shows the difference in the amount of deposited carbon. It seems that the carbon deposit is favored to form at the top of catalyst bed (SR1_Top) where the temperature is high. Meanwhile, the carbon deposition in the reactor #2 are similar. However, the amount of carbon deposits in the reactor #2 is higher than those of the reactor 1, ascribed to the more reactive decomposition of methane.

## Conclusion

In the present work, the upgradation of methane in the biogas by hydrogenation of CO_2_ was investigated in the vertical fixed bed prototype reactor with double pass operation to improve the methane purity and productivity. The Ni-Ce/Al-MCM-41 catalyst which was optimized in the previous study was employed for testing the catalytic performance and the process efficiency under various conditions of feed flow rate, pressure, and temperature as well as durability. As a result, the double pass operation can improve the process efficiency by increasing the purity of biomethane about 15% higher than the single pass operation. Among the several runs with the various parameters, the optimum conditions are determined, including flowrate at 77 ml min^−1^ over 10 g catalyst, pressure at 1 atm, and temperature at 350 °C. Moreover, the long-term stability test shows that the optimized catalyst could perform excellent stability over 458 h of time on stream. Although the larger Ni crystallite size and the carbon deposits were observed in the spent catalyst, the catalyst still exhibits excellent stability with constant high methane productivity. The double pass operation process coupled with the optimized catalyst shows a high potential for the upgradation of methane in the biogas.

## Data Availability

All data related to the finding of this study are accessible upon request from the corresponding author Sakhon Ratchahat.
